# HIV prevalence among children admitted with severe acute malnutrition and associated factors with mother-to-child HIV transmission at Mulago Hospital, Uganda: A mixed methods study

**DOI:** 10.1371/journal.pone.0301887

**Published:** 2024-04-16

**Authors:** Victor Musiime, Joseph Rujumba, Lawrence Kakooza, Henriator Namisanvu, Loice Atuhaire, Erusa Naguti, Judith Beinomugisha, Andrew Kiggwe, Sharafat Nkinzi, Ivan Segawa, Nicholas Matsiko, Esther Babirekere-Iriso, Philippa Musoke

**Affiliations:** 1 Department of Paediatrics and Child Health, School of Medicine, College of Health Sciences, Makerere University, Kampala, Uganda; 2 Research Department, Joint Clinical Research Centre, Kampala, Uganda; 3 Makerere University Lung Institute, College of Health Sciences, Makerere University, Kampala, Uganda; 4 Mwanamugimu Nutrition Unit, Directorate of Paediatrics and Child Care, Mulago National Referral Hospital, Kampala, Uganda; Makerere University School of Public Health, UGANDA

## Abstract

**Background:**

Despite global efforts to eliminate mother-to-child-transmission of HIV (MTCT), many children continue to become infected. We determined the prevalence of HIV among children with severe acute malnutrition (SAM) and that of their mothers, at admission to Mwanamugimu Nutrition Unit, Mulago Hospital, Uganda. We also assessed child factors associated with HIV-infection, and explored factors leading to HIV-infection among a subset of the mother-child dyads that tested positive.

**Methodology:**

We conducted a cross-sectional evaluation within the REDMOTHIV (Reduce mortality in HIV) clinical trial that investigated strategies to reduce mortality among HIV-infected and HIV-exposed children admitted with SAM at the Nutrition Unit. From June 2021 to December 2022, we consecutively tested children aged 1 month to 5 years with SAM for HIV, and the mothers who were available, using rapid antibody testing upon admission to the unit. HIV-antibody positive children under 18 months of age had a confirmatory HIV-DNA PCR test done. In-depth interviews (IDIs) were conducted with mothers of HIV positive dyads, to explore the individual, relationship, social and structural factors associated with MTCT, until data saturation. Quantitative data was analyzed using descriptive statistics and logistic regression in STATAv14, while a content thematic approach was used to analyze qualitative data.

**Results:**

Of 797 children tested, 463(58.1%) were male and 630(79.1%) were ≤18months of age; 76 (9.5%) tested positive. Of 709 mothers, median (IQR) age 26 (22, 30) years, 188(26.5%) were HIV positive. Sixty six of the 188 mother–infant pairs with HIV exposure tested positive for HIV, an MTCT rate of 35.1% (66/188). Child age >18 months was marginally associated with HIV-infection (crude OR = 1.87,95% CI: 1.11–3.12, p-value = 0.02; adjusted OR = 1.72, 95% CI: 0.96, 3.09, p-value = 0.068). The IDIs from 16 mothers revealed associated factors with HIV transmission at multiple levels. Individual level factors: inadequate information regarding prevention of MTCT(PMTCT), limited perception of HIV risk, and fear of antiretroviral drugs (ARVs). Relationship level factors: lack of family support and unfaithfulness (infidelity) among sexual partners. Health facility level factors: negative attitude of health workers and missed opportunities for HIV testing. Community level factors: poverty and health service disruptions due to the COVID-19 pandemic.

**Conclusion:**

In this era of universal antiretroviral therapy for PMTCT, a 10% HIV prevalence among severely malnourished children is substantially high. To eliminate vertical HIV transmission, more efforts are needed to address challenges mothers living with HIV face intrinsically and within their families, communities and at health facilities.

## Introduction

The 2021 World Health Organization (WHO) guidelines for PMTCT, as well as the earlier ones of 2013, recommend universal antiretroviral therapy (ART) for all pregnant and breastfeeding mothers, plus ARV drug prophylaxis for both high risk and low risk infants [1, 2]. When well implemented, these recommendations have a potential to eliminate MTCT. Despite Uganda adopting the guidelines in 2013, children continue to present in health facilities with HIV infection, many with advanced disease [3, 4].

The MTCT rate in Uganda was estimated to be 2.8% in 2021, and 1.5% in 2022 [5, 6]. While this is a significant improvement from 20% in 2000, an estimated 5,500 children acquired HIV in the country through MTCT in the year 2021. It is further estimated that half of these infections occurred among infants whose mothers stopped ART during pregnancy and breast feeding [5, 7]. It is intriguing to understand why these mothers stopped their HIV treatment.

HIV-infected children commonly present with malnutrition and studies done on malnutrition wards have reported high rates of HIV [8–10]. Studies in Mwanamugimu Nutrition Unit, Mulago Hospital in Uganda conducted between 2000 and 2017, have reported a drop in HIV prevalence among the severely malnourished hospitalized children from 44% in 2001, to 24% in 2013, and to 12% in 2017 [11–13]. While this is a marked drop, 1 in 10 children being infected, in this era of universal access to preventive measures including ART, is still a significantly high proportion.

Studies in Uganda and other African countries have reported maternal, infant and health system factors that put children at risk of acquiring HIV from their mothers. The maternal factors include: high viral loads, poor antenatal attendance, suboptimal adherence to ART during pregnancy, delivery and breast feeding, delivery outside health facilities, sexually transmitted infections and malnutrition. The infant factors are: not receiving or poorly adhering to ARVs for prophylaxis, mixed feeding, infant gender and age, while the health system factors include: defective PMTCT services, distant health facilities, understaffing of health facilities and stock outs of essential commodities [14–20].

We therefore set out to ascertain if there is a further change in the prevalence of HIV-infection among children presenting with SAM at Mwanamugimu Nutrition Unit, while determining and exploring the demographic, social and structural factors associated with acquisition of HIV despite availability of PMTCT services. This will be crucial if MTCT is to be eliminated.

## Materials and methods

### Study design

This was a cross-sectional mixed methods study conducted within the REDMOTHIV clinical trial (NCT05051163), from June 2021 to December 2022. The REDMOTHIV trial is a randomized clinical trial, investigating strategies to reduce mortality among HIV-infected and HIV-exposed children admitted with severe acute malnutrition at Mulago hospital [21]. Children aged 1 month to 5 years and their mothers who were available, were tested for HIV on admission. A subset of the mothers also participated in qualitative interviews to explore the factors associated with HIV transmission to the children.

### Study setting

Mulago hospital is a national referral hospital. It is located in Kampala, the capital city of Uganda. The study was conducted in the Mwanamugimu Nutrition Unit of the hospital, which is a specialized center for the management of SAM. It consists of 3 in-patient (stabilization, transition and rehabilitation) wards, an outpatient therapeutic feeding center, a laboratory, a pharmacy and a kitchen. An average of 70 patients were admitted to the unit every month in the year 2022. Mulago hospital is run by the government of Uganda and the services are designed to be provided at no cost to the patient.

### HIV testing

All the children and their mothers were consecutively enrolled and tested with an HIV rapid antibody test using 2 kits in series, namely: DETERMINE^TM^ HIV-1/2 kit from Abbott Laboratories [22] and the HIV-1/2STAT-PAK^®^ kit from CHEMBIO Diagnostic Systems, Inc. [23], following the Uganda Ministry of Health guidelines [4]. This was after obtaining written informed consent from the mothers/ caregivers of the children. The children <18 months of age who tested HIV antibody-positive then had a confirmatory HIV DNA PCR done using a Point of Care assay, the m-PIMA^TM^ HIV 1/2 DETECT from Abbott Laboratories [24]. Majority of the children and a significant number of the mothers, were being tested for HIV for the first time. Post-test counselling was provided to the mothers/ caregivers of the children; and those identified as HIV infected were linked to care at Baylor Uganda COE. The ones that had been diagnosed earlier as living with HIV and were already on ART, continued with it. Those that were HIV-exposed were initiated on cotrimoxazole prophylaxis as applicable, following the Uganda Ministry of Health guidelines [4]. The children that tested HIV-positive or those whose mothers tested HIV-positive were enrolled into the REDMOTHIV clinical trial. All children were then provided with routine care for SAM following the Uganda Ministry of Health guidelines for in patient care for SAM [25].

### Study population

The study participants included children aged 1 month to 5 years admitted at Mwanamugimu Nutrition Unit and their mothers.

#### Inclusion criteria for the quantitative data

- Children aged 1 month to 5 years admitted with SAM at Mwanamugimu Nutrition Unit.
- Mothers of children admitted with SAM at Mwanamugimu Nutrition Unit.
- Mother or guardian providing consent for HIV testing.

#### Exclusion criteria for the quantitative data

- Readmission for management of SAM

#### Inclusion criteria for qualitative data

- Mothers of CLHIV with SAM.
- Provision of informed consent.

#### Exclusion criteria

- Inability to undergo in-depth interviews, for such reasons as illness and unavailability.

#### Study variables

The primary endpoint was the proportion of children that tested HIV-positive, while the secondary endpoint was the proportion of their mothers that tested HIV-positive. The independent variables were: child age, child gender and maternal age.

#### Sample size

We included all the children that were screened for enrolment into the REDMOTHIV trial during the study period (n = 797). This provided us with over 80% power to determine the prevalence of HIV among the children, using that of 12% observed by Nalwanga et al. in Mwanamugimu Nutrition Unit in 2017, as a reference [13].

#### Statistical analysis

The sociodemographic data of the children and their mothers were collected in addition to the HIV test results, and from these the prevalence of HIV among the children and that among the mothers were determined using descriptive statistics. Logistic regression was used to determine the factors associated with HIV infection among the children, considering child age, child gender and maternal age, at bivariate and multivariate analysis. All this was done in STATA version 14 (Stata Corporation, Texas, USA).

#### Qualitative interviews

A subset of mothers of the children who were confirmed to be living with HIV took part in IDIs to further understand the factors that facilitated MTCT. Mothers were enrolled consecutively until there was data saturation. All interviews were conducted by two research assistants trained in conducting qualitative interviews and were audio recorded and later transcribed. The interviews were conducted in the language of preference of the caregiver between Luganda (the local language) and English. The Luganda transcripts were translated into English. On average, interviews lasted 25–40 minutes. Transcripts were cross-checked against audio files by a qualitative researcher. Data was continuously analyzed using a content thematic approach. After 14 interviews no new information was emerging and therefore data collection was ended at interview 16. The content thematic analysis was guided by the Social Ecological Model (SEM) model, which has widely been used in health promotion [26].

#### Ethical considerations

The REDMOTHIV study protocol was approved by the Makerere University School of Medicine Research Ethical Committee (REC. REF. 2020–165) and the Uganda National Council of Science and Technology (REF. HS1277ES). Regulatory approval was obtained from the Uganda National Drug Authority (NDA) (REF. CTC. 0168/2021). Administrative clearance was obtained from Mulago National Referral Hospital (REF. MHREC. 1942). All mothers of the participating children provided written informed consent, using the consent documents in English and Luganda. The data collected was kept under lock and key, and saved in a secure database.

## Results and discussion

A total of 797 children and 709 mothers were tested for HIV. Most of the children (79.1%) were <18 months of age, and a slight majority were male. The median age (IQR) of the mothers was 26 (22, 30) years. The demographics of the children and the mothers are summarized in Table 1.

**Table 1:** Participant characteristics.

**Child gender** **(N = 797)**	Male– 463 (58.1%)
	Female– 334 (41.9%)
**Child age (months)** **(N = 797)**	Median (IQR)– 12 (7,18)
	≤ 18 months– 630 (79.1%) > 18 months– 167 (20.9%) (19–60 months)
**Mother age (years)** **(N = 709)**	Median (IQR)– 26 (22,30)
	≤24 years– 293 (41.3%) >24 years– 416 (58.7%)
**Child HIV Prevalence** **(N = 797)**	9.5%
**Mother HIV Prevalence** **(N = 709)**	26.5%

Of the 76 children that tested HIV positive, 66 had their mothers available and also tested. Since 188 mothers tested positive, this implies an MTCT rate of 35.1% (66/188), in this population, which is very high.

### HIV prevalence and associated factors

The prevalence of HIV among the severely malnourished children was 9.5%, while that among the mothers was 26.5% (Table 1). Child age >18 months (19–60 months) was marginally associated with HIV-infection (crude OR = 1.87,95% CI: 1.11–3.12, p-value = 0.02; adjusted OR = 1.72, 95% CI: 0.96, 3.09, p-value = 0.068). Child gender and mother age group were not observed to be associated with HIV infection, as shown in Table 2.

**Table 2:** Factors associated with HIV transmission.

Factor	Crude OR (95% CI)	p-value	Adjusted OR (95% CI)	p-value
**Child gender**				
Male	1	0.779	1	0.727
Female	1.07 (0.67,1.73)		0.90 (0.51, 1.61)	
**Child age**				
≤ 18 months	**1**		**1**	**0.068**
>18 months (19 – 60months)	**1.87 (1.11,3.12)**		**1.72 (0.96–3.09)**	
** Mother age**				
≤ 24 years	1	0.169	1	0.731
>24 years	0.61 (0.31, 1.23)		1.13 (0.55, 2.37)	

#### Exploration of the factors associated with HIV infection among the children

Sixteen mothers participated in qualitative interviews. Their age ranged between 19 and 45 years with a mean (± standard deviation) age of 29.5 (± 6.9) years. Regarding education, half of the mothers had attained primary education, 7/16 secondary education, with only one having attained tertiary education. Half of the study participants were married/living with a partner at the time of the study, while 5/16 had separated. Overall 9/16 were unemployed at the time of the study while 7/16 were employed in the informal sector. Most mothers (14/16) were on ART compared to 10/16 of their children. The findings were grouped in line with the Socio-Ecological Model (SEM) [26], as individual, relationship, health facility and community level factors, as summarized in Table 3.

**Table 3:** Thematic presentation of factors associated with child HIV infection.

Theme/Level	Sub-theme- risk factor
Individual	• Inadequate information on mother to child transmission of HIV • Limited perception of HIV risk • Fear of antiretroviral drugs
Relationship	• Lack of family support • Unfaithfulness (Infidelity) among sexual partners
Health system	• Negative attitude of health workers • Missed opportunities for HIV testing
Community	• Poverty • Health service disruption due to the COVID-19 pandemic

#### Individual level factors

*Inadequate information on mother to child transmission of HIV and limited perception of HIV risk*. Some mothers revealed limited awareness about mother to child transmission of HIV. Others doubted their own HIV status and the need for ARVs to prevent HIV infection to their babies, especially those that had given birth to HIV-negative children before. Such mothers did not initiate or adhere to ARVs which facilitated HIV transmission to their children.

*I didn’t think about it because the ones (children) I had before, I had them when I was infected but they were negative (Mother 14, 45-year-old)*

Some mothers administered ARVs to their babies for a short time and yet they continued to breastfeed. Practicing mixed feeding was another indicator of inadequate knowledge among study participants regarding mother to child transmission of HIV and was mentioned as an associated factor of HIV infection in children, as one mother explained.

*I used to give him (child) breast milk and tea…maybe he got HIV through breastfeeding. Because remember, when you give tea, the child might get some small wounds yet from there, you put him on the breast. Remember I didn’t know anything … (Mother 15, 33-yea.r-old)*

*Fear of antiretroviral drugs*. Some mothers feared the side effects of ARVs and thus did not initiate the drugs or give them to their babies which facilitated the acquisition of HIV.

*I started taking the drugs (ARVs) but after I stopped and I feared to resume taking them. I was told that I would die if I did (Mother 2, 20-year-old)*.

*I was initiated on ARVs but the boy was not because I thought he was too weak to start ARVs. He was just born (Mother 5, 31-year-old)*

The fear for ARVs was reinforced by rumors. These included a belief that ARVs would quicken mothers’ death or all HIV-exposed children automatically acquire HIV from mothers. Such myths and rumors discouraged women from adhering to ARVs, and giving babies ARVs as one mother explained.

*I hear that pregnant women die faster with HIV…so I stopped them (ARVs). I carried my pregnancy without taking those drugs. Even my husband did not know because it was just a battle between me and myself. So, I knew at some point, maybe I would die at delivery, may be something would just happen to me and I drop dead. I gave birth and whispered to the nurse that “I am positive and I have not been on drugs”. So, the nurse gave the baby nevirapine. I was like now that I walked the journey of pregnancy without taking drugs, this baby must be infected. Why am I even struggling? I ended up breastfeeding him, and I just stopped everything (Mother 5, 31-year-old)*

#### Relationship level factors

*Lack of family support*. Lack of support from male partners and other family members hindered women from accessing PMTCT services. In some instances, where women accessed ARVs for their own health and for PMTCT, lack of support from male partners discouraged women from using the medicines thus increasing the chances of their babies acquiring HIV.

*I had some medication at home with me by the time I made two months of being pregnant. But from two months up to the end of the 9 months, I wasn’t taking any medication because my husband wasn’t taking care of me (Mother2, 20-year-old)*

Another mother who was born with HIV defaulted on ART during pregnancy, delivered from home, and the baby did not get ARVs because of lack of partner and family support.

*My husband was not taking good care of me. He (husband) knew that I was HIV positive but never used to care. Each time I would tell him that I had to go to hospital, he would tell me that he had no money. I also tried to get to my husband’s parents to intervene but they also never minded and instead they always sided with their son. I didn’t go to hospital, gave birth from home and the child did not get nevirapine … (Mother 13, 19-year-old).*

One woman in search for support, got a new sexual partner during the breastfeeding period but did not test for HIV and this exposed the child to HIV infection.

*After giving birth, me and my partner were not together and I was there just like you know personal things, you might be there when you need some help, like what to eat as you know but when you don’t have. At that moment I got a boyfriend and we did not use any protection, we did not test first, … I thought that was the source of problems (HIV infection) because I was still breastfeeding (Mother 1, 20-year-old).*

*Unfaithfulness (Infidelity) among sexual partners*. Other women linked their acquisition of HIV to the infidelity of their male sexual partners. This way they transmitted this acquired HIV to their babies, as one mother narrated.

*What I suspect, may be the child’s father later contracted the virus and then he infected me. He was never at home. He would go for work and come back home (Mother 4, 24-year-old).*

Such a mother was unlikely to be aware of her status before and hence never sought PMTCT services, given that she was only speculating how she may have acquired the infection.

#### Health system factors

*Negative attitude of health workers*. Mothers revealed that some health workers were rude and unfriendly which discouraged women from using the PMTCT service package, thus exposing children to HIV infection. Some actions of the health workers did not take into consideration of the special circumstances the mothers were in, as narrated by this mother.

*The medical personnel asked me where I was picking my ART medication from and wrote for me an antenatal card. They said that much as I wanted them to help me deliver, they didn’t know the amount of virus in my blood, and so, they tore my antenatal card, yet I had no money to bring me to Kampala (Mother 2, 20-year-old).*

*Missed opportunities for HIV testing and re-testing at delivery and during breastfeeding*. Four mothers mentioned that they tested HIV negative as part of antenatal care and were not re-tested during pregnancy, delivery or breastfeeding until when their children started falling sick or were malnourished and admitted at the nutrition unit. This indicates missed opportunities for HIV testing as an entry point to HIV care and prevention.

*They tested me when I was pregnant and told me that I was HIV negative. If only I had gotten tested again and got to know, I would have started ARVs. Nothing was done because I didn’t think I was infected (Mother 15, 33-year-old)*.

*Not testing for HIV. If I had known my HIV status and started treatment early enough, my child wouldn’t have contracted the virus. Even if me the adult would be positive but at least my child would be safe (Mother 10, 38- year- old)*.

*I delivered from a private hospital and that’s where I have delivered all my children. The nurse was there but they didn’t test me …(Mother 3, 29- year- old).*

#### Community level factors

*Poverty*. Study participants mentioned challenges of poverty that hindered access to health facilities for maternal and child health services including PMTCT services. Some mothers had delivered at home or attended fewer antenatal visits due to financial constraints.

*I had a lot of problems and couldn’t support myself. I decided to stay home and be strong and I ended up delivering from home. I lacked support in terms of money to take me to hospital (Mother 14, 45 -year-old)*

*I was supposed to correctly take my medication properly but I never used to get the medication, I didn’t have money for transport… (Mother 13, 19- year- old).*

*Disruptions in access to health services due to the COVID-19 pandemic*. Some mothers linked HIV infection in their children to disruptions in access to HIV care and prevention services due to COVID-19 and related movement restrictions. One mother narrated how she relocated to a village in the rural areas during the COVID-19 pandemic and failed to access ARVs from the village health facility. Her usual ART refill health centre was in the urban setting where she stayed before the COVID-19 movement restrictions put her out of work and a livelihood.

*My ARVs got finished while I was far from my usual health centre where I get care from. I went to another health centre to get medication and they (health workers) sent me away that they need a transfer letter; which I couldn’t get because I had no money for transport… (Mother 2, 20-year-old).*

Another one added:

*COVID-19 really affected us in terms of transport. We had no transport means to go and pick treatment (Mother 11, 32-year-old).*

Another defaulted on her medication:

*When I went to the village during the COVID-19 period, I defaulted on medication…I used to get my treatment…I feared going back to the hospital because the doctor would have abused me for having defaulted on medication (Mother 11, 32-year-old).*

### Discussion

Children continue to present in health units with HIV infection, many with advanced disease, despite the local, regional and global efforts for PMTCT. In this study we found that 1 in 10 severely malnourished children were living with HIV at admission to Mwanamugimu Nutrition Unit, Mulago Hospital. We observed a very high MTCT rate of 35%. The study identified child age, as well as several social and structural factors at multiple levels to be associated with HIV transmission among these children.

Studies done earlier at Mwanamugimu Nutrition Unit showed a drop in the HIV prevalence among the children admitted with SAM [11–13]. The sharper drop between 2013 and 2017 is most probably explained by the 2013 WHO recommended Option B plus strategy for PMTCT which advocates for universal ART for pregnant women and during breastfeeding as well as infant ARV prophylaxis, [1] which Uganda adopted. Contrary to this trend, the drop from 12% in the 2017 study [13] to the 9.5% we observed in this study is not as marked, in spite of the WHO option B plus strategy still being followed. This study has identified several probable explanations.

From the IDIs, mothers reported lacking knowledge on PMTCT as well as having several myths and rumors on ARVs. A study on barriers to PMTCT in the Eastern Cape, South Africa also identified inadequate knowledge as a barrier [27]. This knowledge gap needs to be bridged.

The mothers also reported lack of family support as a factor, either with resources to access PMTCT services or with moral support to adhere to medication provided or even remain in care. An earlier study in Uganda, conducted in Wakiso district which surrounds Kampala city where Mulago Hospital the site for our study is located, reported that access to PMTCT services was better when male partners offered support to HIV positive women [28].

Similarly, the mothers reported factors in their communities that were a barrier to accessing care. The need for finances for transportation to health facilities to access PMTCT services can’t be over emphasized. Many of the mothers that participated in this study, and indeed from malnutrition units in Uganda and elsewhere, are from poor families. Even when they knew what to do, the lack of money prevented them from accessing care. This too was reported to be an issue in the earlier Ugandan study [28]. It is possible that if PMTCT services were brought to nearer, distance as a barrier may be addressed, as the study in the Eastern Cape, South Africa implied [27]. The downside to this is that nearby HIV clinics are often associated with stigma, a key barrier to PMTCT [29]. It may be worthwhile to consider financial incentives and other economic approaches for pregnant women and breast-feeding mothers living with HIV, as suggested by a recent study in South Africa [30].

The problem of failure to access PMTCT services because of lack of family support and by socioeconomic challenges was compounded by disruptions brought about by movement restrictions during the COVID-19 pandemic. Indeed, a study done in HIV care clinics in Kampala, showed that retention in care was adversely affected by the COVID-19 pandemic [31]. This calls for epidemic preparedness to prevent disruptions in health service delivery.

Several health system factors were reported by mothers, key among which were missed opportunities for HIV testing and the negative attitude of health workers. It is possible that understaffing or staff changes in the health facilities or shortages in testing kits, may have been contributory, like earlier reported by a study in rural Uganda [15]. Such gaps in the health systems coupled with socioeconomic challenges, prompt mothers to deliver from home or facilities without adequate PMTCT services, as was reported by mothers in our study. Deliveries from such settings have been reported to be associated with HIV transmission to babies in Uganda and Ethiopia [14, 15, 17]. This underscores the need for the PMTCT program in Uganda to address barriers that are specific to the health system.

In this study we observed that child age over 18 months was marginally associated with HIV infection. While the children over 18 months of age contributed to only 20% of the study population, this study suggests that this age group of severely malnourished children need to be studied further. A large longitudinal study in Malawi among HIV exposed children showed that HIV prevalence increased by age of the HIV exposed child [32]. A similar study in rural Kenya showed that child age over 6 months at enrolment was associated with HIV infection [16]. It is possible that many of these older children acquired HIV through breastfeeding from mothers who were either unaware of their HIV status and were therefore not on ART, or mothers who had concurrent illnesses. In our study, several mothers got to know their HIV status in the study when their children were concurrently tested. In the Kenyan study, the mothers that were not on ART were more likely to transmit HIV to their children [16]. It may therefore be necessary to put special emphasis on older children on malnutrition wards, with regard to HIV testing and other HIV services. Nevertheless, younger children (under 18months of age) need not be neglected for the same services.

The MTCT rate in this population of mothers living with HIV at 35% was very high when compared to the overall national rate of 1.5% [6]. While we did not assess the mothers’ nutritional status and their food security, maternal and infant malnutrition as well as household food insecurity may have had a part to play, given that they have been associated with higher chances of MTCT [33, 34]. The relationship between increased chances of MTCT and both maternal and infant malnutrition is likely due to micronutrient deficiencies (of vitamins A, B-complex, C, D, E and selenium), which facilitate HIV transmission [33]. On the other hand, household food insecurity has been reported to be a barrier to uptake of such PMTCT services as antenatal care attendance, knowing one’s HIV status, facility based delivery and postnatal visit attendance [34]. In addition, the psychosocial factors described by the mothers and discussed here, such as poverty and other socioeconomic challenges, are probably responsible for poor access to PMTCT services and hence this high MTCT rate, and they possibly disproportionately affect families with malnourished children. Furthermore, while the health of the mothers was not assessed, it is possible that many of the mothers had unsuppressed viral loads, making it easier to transmit HIV, especially through breastfeeding [16]. Indeed, many of the mothers only became aware of their positive HIV status when they were screened for this study and were therefore not yet on ART. The large proportion of HIV exposed (infected and uninfected) children in this nutrition unit, reflected by the proportion of the mothers that tested positive (approximately 1 in 3) underscores the vulnerability of these children with regard to development of severe acute malnutrition [10, 35].

In the quantitative analysis, we were only able to assess three factors that could have been associated with HIV transmission; child gender, child age, and maternal age, because this was the only data that was collected on the screening form for the REDMOTHIV trial. Despite this limitation, we were able to explore other possible factors in the qualitative interviews. The strength of the study therefore was in its mixed methods approach. Another key limitation is that, in this population of severely malnourished children in the context of MTCT, we were not able to collect data on the mothers’ nutritional status and their knowledge and understanding of their own and their children’s nutritional needs.

## Conclusions

The prevalence of HIV among children with SAM is still substantially high, therefore routine HIV testing in this population is still warranted in Uganda and similar settings. The healthcare providers working on malnutrition wards in these settings will need to recognize that they continue to have a large number of mothers living with HIV as attendants, and consequently a large number of HIV exposed and HIV infected children who have to be identified and linked to care. As the global community aims to eliminate MTCT of HIV, measures tailored to address specific health system and community bottlenecks must be employed. Mothers living with HIV will need to be empowered socioeconomically and with current knowledge on PMTCT, so that they are able to seek antenatal care early and optimally utilize PMTCT services. This will require that ministries of health work hand in hand with all stakeholders to disseminate HIV prevention messages in a sustained way, while ensuring that HIV testing kits and ARVs are constantly in stock at the facilities. The health facilities should be easily accessible, with services/ outreaches at community level and with adequate numbers of empathetic and knowledgeable healthcare providers regarding PMTCT. The high MTCT rate we observed in this population coupled with the well documented relationship between HIV and malnutrition suggests that there may be a role of such nutritional interventions as micronutrient supplementation and ensuring household food security in PMTCT.
